# Location, location, location: Protein kinase nanoclustering for optimised signalling output

**DOI:** 10.7554/eLife.93902

**Published:** 2024-01-11

**Authors:** Rachel S Gormal, Ramon Martinez-Marmol, Andrew J Brooks, Frédéric A Meunier

**Affiliations:** 1 Clem Jones Centre for Ageing Dementia Research, Queensland Brain Institute, The University of Queensland Brisbane Australia; 2 Frazer Institute, The University of Queensland Woolloongabba Australia; 3 School of Biomedical Sciences, The University of Queensland St Lucia Australia; Goethe University Germany; Goethe University Germany

**Keywords:** protein kinase, nanocluster, super-resolution microscopy, signalling

## Abstract

Protein kinases (PKs) are proteins at the core of cellular signalling and are thereby responsible for most cellular physiological processes and their regulations. As for all intracellular proteins, PKs are subjected to Brownian thermal energy that tends to homogenise their distribution throughout the volume of the cell. To access their substrates and perform their critical functions, PK localisation is therefore tightly regulated in space and time, relying upon a range of clustering mechanisms. These include post-translational modifications, protein–protein and protein–lipid interactions, as well as liquid–liquid phase separation, allowing spatial restriction and ultimately regulating access to their substrates. In this review, we will focus on key mechanisms mediating PK nanoclustering in physiological and pathophysiological processes. We propose that PK nanoclusters act as a cellular quantal unit of signalling output capable of integration and regulation in space and time. We will specifically outline the various super-resolution microscopy approaches currently used to elucidate the composition and mechanisms driving PK nanoscale clustering and explore the pathological consequences of altered kinase clustering in the context of neurodegenerative disorders, inflammation, and cancer.

## Introduction

Protein kinases (PKs) are important enzymes that play critical cellular roles by controlling cell signalling and a myriad of associated functions. They act by catalysing the phosphorylation of specific target proteins, thereby modulating their activity, subcellular localisation, and function. Through protein phosphorylation, kinases orchestrate essential cellular processes such as cell growth, differentiation, metabolism, and intricate signal transduction. Their mode of action relies on the catalytic transfer of a phosphate group from adenosine triphosphate (ATP) to their selective target proteins (Ubersax and Ferrell, 2007). This phosphorylation acts as a switch in their function by initiating a cascade of molecular events controlling selective cellular behaviours (Taylor and Kornev, 2011). Following binding to the catalytic domain of the kinase, ATP is converted to adenosine diphosphate (ADP) and an inorganic phosphate (Pi) which is covalently transferred to a protein substrate, leading to the modification of its activity (Cohen, 2001). PKs exhibit a high degree of diversity, with various ways to classify them (Modi and Dunbrack, 2019) based on their specific structural motifs (eukaryotic/atypical) (Kanev et al., 2019) and target amino acid residues. The three main classes of PKs are serine/threonine protein kinases (STPKs), tyrosine kinases (TKs), and dual specificity protein kinases (DSPKs) (Fabbro et al., 2015).

STPKs represent most of the eukaryotic PKs. As indicated by their name, they act by phosphorylating serine and threonine residues in their substrate/target protein. These kinases are involved in regulating various cellular processes, including cell growth, proliferation, differentiation, and apoptosis. STPKs play critical roles in signalling pathways, such as the transforming growth factor-beta pathway and the mitogen-activated protein kinase (MAPK) pathway. They can be further subdivided into ‘classical’ STPKs and atypical STPKs that are only found in certain cell types.

TKs constitute a distinct class of PKs that selectively phosphorylate tyrosine residues. TKs play crucial roles in cellular communication, proliferation, differentiation, and survival. They can be further categorised into receptor-associated TKs, which participate in receptor signalling pathways, and non-receptor-associated TKs, which interact with DNA in the nucleus. Prominent examples of TKs include the epidermal growth factor receptor (EGFR), the insulin receptor, and the JAK, and Src family kinases (SFKs).

DSPKs possess the unique ability to phosphorylate both serine/threonine and tyrosine residues. These kinases exhibit versatility in their substrate specificity and often participate in complex cellular processes. DSPKs are involved in regulating cell cycle progression, DNA damage response, and cellular stress signalling.

PKs can also be classified based on their specific functions into signalling PKs, metabolic PKs, and housekeeping PKs. Signalling PKs are involved in signal transduction, metabolic PKs regulate cellular metabolism, and housekeeping PKs perform essential functions within the cell.

### PK nanoclustering: implications for signalling

**Figure 1.**
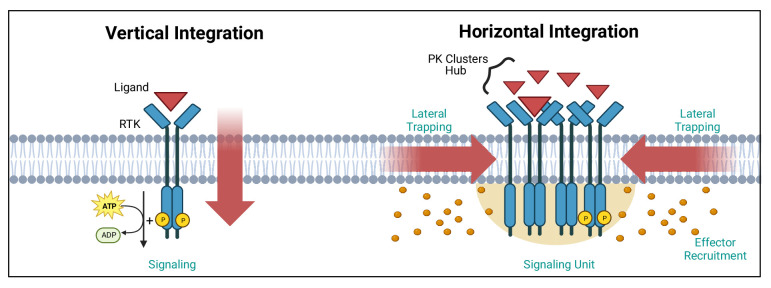
Vertical and horizontal integration of the signal. Protein kinase (PK) activation is classically described as the result of an extracellular ligand (red triangle) binding to a plasma membrane receptor (e.g. receptor tyrosine kinase [RTK]) and activating PKs and downstream effectors (orange circles). This vertical integration of the signal has recently been refined to include horizontal integration with the realisation that some PKs organise into nanloclusters. At the plasma membrane, this horizontal integration occurs via lateral trapping and nanocluster formation of inherently diffusible receptors (and their associated PKs), which gives rise to signalling ‘hubs’ or ‘units’. We have exemplified these two concepts using RTKs. Created with https://www.biorender.com/.

The subcellular organisation of PKs is of great importance for their signalling function. Our understanding of signalling mainly revolves around a vertical integration of the signal, with ligand binding to receptor, receptor activation and PK initiation of the signalling cascade (Figure 1). With the super-resolution microscopy revolution in cell biology, came the realisation that PKs are organised in clusters with sizes below the diffraction limit of light (Martínez-Mármol et al., 2023a; Owen et al., 2010; Padmanabhan et al., 2019). The mechanism(s) underpinning such clustering and its functional outcome for PK activity is/are still open questions, but the emerging concept points to a horizontal integration of the signal revolving around the lateral trapping of receptors and associated PKs in nanoclusters (Figure 1). Upon binding of the ligand, these nanoscale hubs can, in turn, generate hubs of PKs and of other downstream effectors raising the possibility that nanoclusters could serve as quantal units of signalling function. Such horizontal organisation of receptors and their effectors creates dynamic discrete signalling hubs, likely relying on structural pro-clustering sequences and interfaces leading to dimerisation and oligomerisation. This may involve the non-catalytic PK (or association of pseudokinases), as well as post-translational modifications (PTMs; e.g. phosphorylation) controlling these interactions. Understanding the modality of PK clustering will require multidisciplinary approaches to grasp how the output signal is integrated both vertically and horizontally in space and time to generate a downstream functions.

Due to the large variety of PKs, this review will focus primarily on a limited number of PKs that have been studied with super-resolution microscopy. We will also attempt to draw a roadmap detailing the use of super-resolution microscopy to assess the nanoscale organisation of PKs. We will first discuss the main advantages of using sub-diffraction microscopy to assess the dynamics of PK nanoscale organisation, and how this can alter protein signalling cascades in health and disease. We will also briefly cover the main technologies used in these studies. We will then discuss PK membrane clustering, cytoplasmic re-organisation of clustering mediated by lipidation and biomolecular condensates. The review will finally explore the ramifications of alterations in PK clustering in neurodegeneration and cancer.

### Advancing PK research with super-resolution microscopy

**Figure 2.**
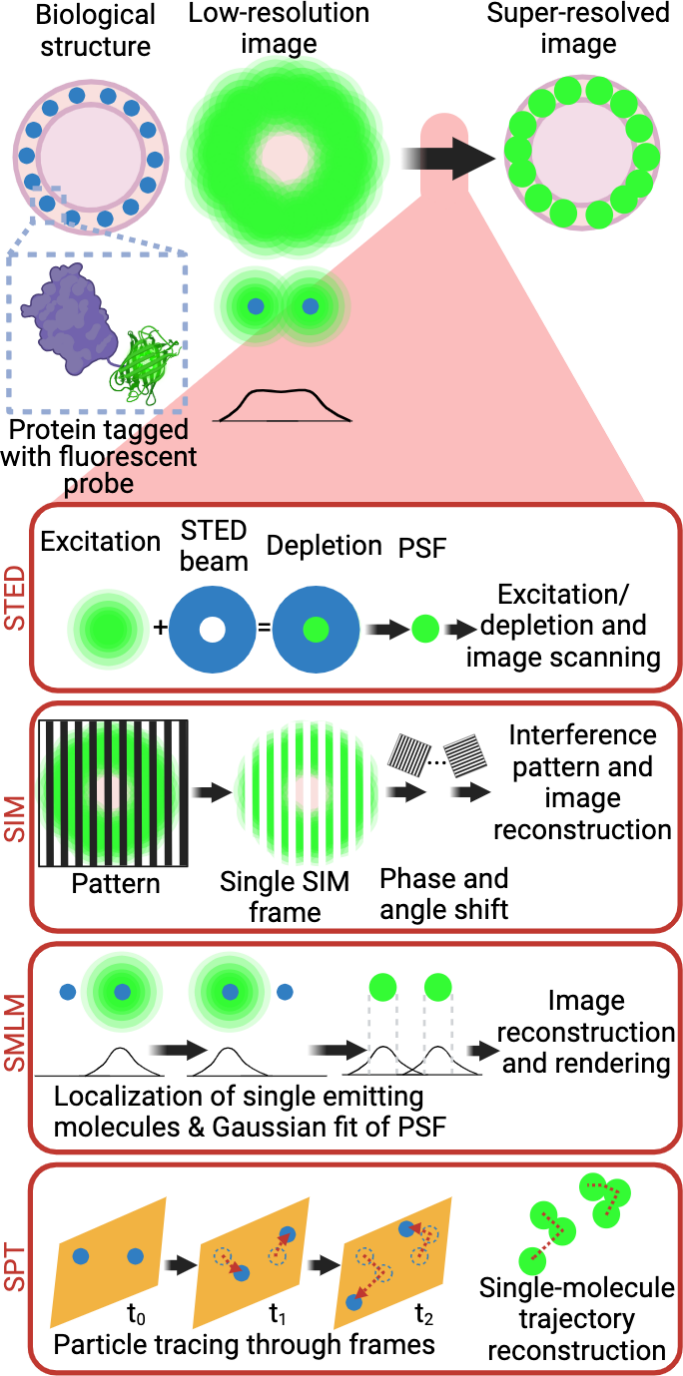
Super-resolution microscopy techniques. Selective labelling of proteins with antibodies or tagging with fluorescent proteins allowed their direct visualisation by light microscopy. However, the identification of small cellular structures has been limited by the diffraction limit of light. Only the implementation of super-resolution imaging efficiently solved this limitation. There are three main super-resolution approaches. In STED microscopy, the focused excitation light is combined with a depletion doughnut-shaped beam, decreasing PSF size to a volume smaller than the diffraction limit. Scanning the sample with an excitation light aligned with STED light beam allows the creation of super-resolved images. In SIM, the sample is imaged with a grid-like pattern of light. The interference patterns between the sample and the illumination grid create Moiré fringes. Multiple images are obtained with varying phase shifts of the patterned light, which are used to reconstruct a sub-diffraction image. In SMLM, the precise position of individual emitting molecules is obtained by fitting their intensity profile to a Gaussian model of the PSF. Acquisition of single localisations depends on the low density and stochastic excitation of emitters. Single localisations are then combined to reconstruct the super-resolved image. In live cells, SPT, single-molecule trajectory reconstructions can be achieved by tracking the individual detections of a single fluorophore across the acquisition window in living samples. *Abbreviations*: STED, stimulated emission depletion; PSF, point spread function; SIM, structured illumination microscopy; SMLM, single-molecule localisation microscopy; STP, single-particle tracking. Created with https://www.biorender.com/.

Fluorescence microscopy has been instrumental in revealing the subcellular localisation of most PKs, providing additional information on their signalling functions. However, the relatively low resolution of fluorescence microscopy limits our ability to address critical questions inherent to the nanoscale environment in which they operate. The resolution of light microscopy is constrained by the diffraction limit of light, which depends on the wavelength of the illumination light used (typically ~250 nm). Furthermore, many hundreds, if not thousands, of molecules are needed to achieve a signalling output in discrete subcellular locations. In such inherently crowded nano-environments, the fluorescence of so many emitters largely overlaps, leading to the detection of large blobby structures lacking spatial resolution. To some extent, super-resolution microscopy can overcome the diffraction limit of light. There are three commonly used types of super-resolution microscopy techniques (Figure 2) including stimulated emission depletion (STED) (Heine et al., 2018), structured illumination microscopy (SIM) (Gustafsson, 2008), and single-molecule localisation microscopy (SMLM) (Betzig et al., 2006; Rust et al., 2006; Heilemann et al., 2008).

Common SMLM approaches include, stochastic optical reconstruction microscopy (STORM), photo-activated localization microscopy (PALM) as well as point accumulation for imaging in nanoscale topography (PAINT). Regardless of the methods used, the output of SMLM is a list of co-ordinates of each single-molecule’s detection. Spatially separated localisations are achieved for each approach by either imaging organic dyes in a reducing buffer to induce blinking (STORM), by visualising photoconvertable/switchable proteins applying targeted lasers (PALM), or by incubating with fluorescent binding probes that can bind reversibly or at sparse concentrations (PAINT) (Godin et al., 2014). SMLM uses the point spread function (PSF) of single fluorescence emitters to fit a Gaussian function and establish the Cartesian coordinates of each emitter with sub-pixel precision, depending on the method used. The PSF is usually circular in the two dimensions (*x*, *y*) and elliptical in depth (*z*), and its size depends on the wavelength of the light and the numerical aperture of the objective lens. By fitting a Gaussian function to the PSF, the localisation of the fluorophore can be estimated with relatively high accuracy. Many excellent reviews describe the pros and cons of each super-resolution technique (Valli et al., 2021; Mahecic et al., 2019; Cox, 2015). One of the key advantages of SMLM is that it can be used both in fixed and live cells (Manley et al., 2008) and is amenable to tracking endogenous proteins via a range of methods including intrabody expression of selective single-chain antibodies (e.g. camelid nanobodies) (Gormal et al., 2020) or CRISPR/Cas9-based endogenous tagging (Willems et al., 2020). They require specialised tags and rely on the activation of a photoswitchable fluorophore that emits sufficient photons for reliable localisation before becoming bleached or going into a dark state (Lelek et al., 2021). When this is used to track molecules over several consecutive images, it is called single-particle tracking (SPT) and can be used to derive mobility measurements such as the diffusion coefficient (Gormal et al., 2020). Since SPT computes the mobility of molecules as they perform their actions, some important temporal metrics can be obtained. Applying specialised analysis methods to SPT data can capture single-molecule dynamics, including diffusion, active transport, and spatiotemporal clustering (Cho et al., 2016; Wallis et al., 2023). One of the drawbacks of photoactivatable, photoswitchable, and photoconvertible tags is that the trajectories generated are relatively short. Depending on the requirement of the experiments, other techniques based on self-labelling enzymatic tags can be used, such as SNAP tags and Halo tags that are also genetically fused to the target protein. They covalently bind their bright fluorescent cell-permeant ligands (Lelek et al., 2021). The sparse labelling obtained with these techniques can be used in fixed and live cells. In the latter case, long trajectories can be generated with high localisation precision. Classically, SMLM techniques have relatively good localisation precision in the range of 10–40 nm (Figure 2), but new technologies can achieve higher spatiotemporal resolutions (Fujiwara et al., 2023; Deguchi et al., 2023). STED microscopy uses a different approach to achieve improved localisation precision by reducing the size of the PSF. STED employs two super-imposed laser beams with different excitation patterns, that raster-scan the entire sample, pixel by pixel. Since the second laser beam can de-excite the periphery of the excited fluorophore, the resulting emission is much smaller (Hell and Wichmann, 1994; Vicidomini et al., 2018). For live imaging experiments, SIM provides a direct approach that can achieve super-resolved images with conventional fluorescent microscopy sample preparation. By applying the light to the sample through known patterns at different lateral positions (rotating), emissions from out of focus light can be removed during post-processing, achieving super-resolved images with an improved lateral resolution of ~150 nm (Gustafsson, 2000). Since both SIM and STED both remove photons from the imaging sample, it is critical that the samples are sufficiently bright to achieve the best results. Overall, a range of super-resolution techniques are well suited to the study of PKs, providing a better understanding of the relationship between their spatiotemporal localisation and the signalling generated. These sub-diffractional methods provide an opportunity to characterise the organisation of PKs into discrete clusters, to varying degrees, by applying tailored analytical approaches (Chenouard et al., 2014; Khater et al., 2020). For instance, fixed STED and SMLM approaches can identify metrics such as cluster size and density, whereas SPT approaches can provide information on other temporal aspects such as mobility and time spent immobilised within hotspots.

### Thermodynamic considerations on PKs localisation and their signalling output

Many PK-mediated signalling processes depend on their ability to phosphorylate their substrates. How they access their substrate in a timely fashion is therefore critical to the efficiency and timing of the phosphorylation process. One of the main hindrances to the timely access of PK to substrate comes from Brownian thermal energy, which generates a chaotic nanoscale environment tending to homogenising both PK and their substrates in the entire volume of the cell. The diffusion of an average-sized protein in cellulo is approximately 10 μm^2^/s. This means that cytosolic proteins can navigate the entire length of a cell within a few seconds bouncing against other proteins randomly (Elowitz et al., 1999). The ability of PKs and their substrates to efficiently react in space and time must require some level of immobilisation. This is a critical factor to consider in the timing and integration of the output signal. In this context, it is not surprising that PK have been found to be immobilised in small sub-diffractional clusters. Nanoclustering of plasma membrane receptors by transient lateral trapping is emerging as a novel mechanism for efficient and selective signalling (Liang et al., 2018; Harding and Hancock, 2008). The affinity and accessibility of PKs to their target substrates are enhanced by their concentration, and therefore the mechanism(s) controlling the nanoscale organisation of PKs in clusters and that of their substrate is of great interest to the field. In addition, the role of receptor–ligand binding on transphosphorylation, hetero-/homo-dimeric/oligomeric re-organisation as well as allosteric competition between downstream effectors likely further specialises the downstream response as recently shown (Chhabra et al., 2023). It is important to note that kinase signalling is not solely reliant on phosphorylation of substrates (Jacobsen and Murphy, 2017). For instance, pseudokinases, which have little to no catalytic activity and make up about 10% of the kinome, are integral components of many signalling pathways (Kung and Jura, 2016; Patel et al., 2020).

Several mechanisms are therefore hypothesised to mediate PK and pseudokinase immobilisation into hubs that influence downstream signal propagation. This includes the ‘fences and pickets’ plasma membrane model. In this model, receptor PKs are compartmentalised by actin-based membrane-skeleton ‘fences’ and anchored transmembrane protein ‘pickets’, clustering them in space and time at the plasma membrane (Kusumi et al., 2014). Protein nanodomains can also be driven by intra- and inter-molecular interactions including oligomerisation (Sieber et al., 2006). Prior to super-resolution approaches, the examination of PK organisation and its effect on cellular signalling mainly relied upon spatial techniques (Owen et al., 2010) leaving the temporal aspect of clustering unresolved. As such, the contribution of clustering and the role that lateral trapping plays in the activity of cell-surface receptors and other PKs, remains to be elucidated.

### Dynamics of RTK clustering at the plasma membrane

**Figure 3.**
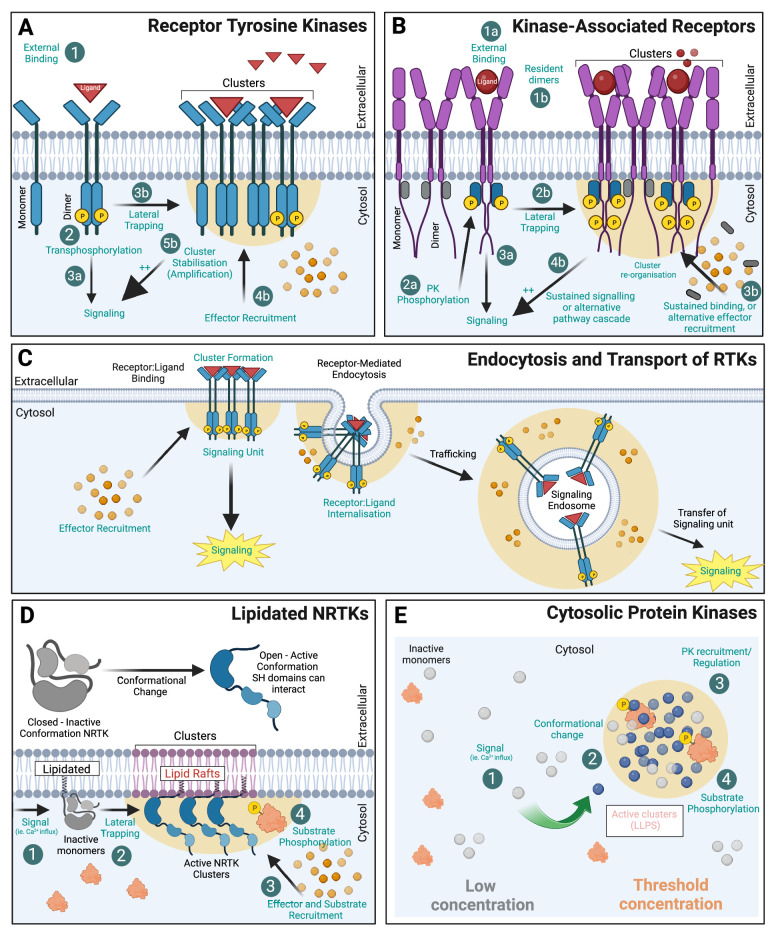
Protein kinase (PK) clustering mechanisms. (**A**) Receptor tyrosine kinases (RTKs) with intrinsic kinase activity exist at the membrane as both monomers and dimers. Upon ligand binding, trans-phosphorylation of the receptor initiates downstream signalling cascades. The formation of receptor kinase clusters (by lateral trapping) also promotes associated hubs of ligands (red triangles) and effectors (orange circles) that amplify the signal. (**B**) Kinase-associated receptors (e.g. cytokine receptors) rely on non-receptor PKs to associate to their intracellular domains and activate signalling cascades. Many act through dimerisation (1 ligand:2 receptor) and induced conformational change within their intracellular domain (ICD) allowing PK transphosphorylation (grey 'inactive’, blue 'active’) and subsequent effector recruitment. (**C**) RTKs undergo receptor-mediated endocytosis and endocytic trafficking. Signalling endosomes can continue to signal during transport thereby transferring the ‘signalling hub’ to its subcellular destination. (**D**) Non-receptor tyrosine kinases (NRTKs) can be post-translationally modified via lipidation (myristoylated or palmitoylated) allowing association with membranes. Many of these PKs are allosteric in nature and require a signal (such as Ca^2+^) to alter their conformation and allow subsequent interaction with their substrates. For many, N-terminal myristylation mediates PK association to lipid rafts where they are more active. (**E**) Cytosolic PKs can also compartmentalise into biomolecular condensates (BMCs) which are membraneless compartments, formed via liquid–liquid phase separation (LLPS). Created with https://www.biorender.com/.

Unique to PK-linked receptors is that their intracellular catalytic activity can be triggered in response to an external ligand (Figure 3A). Receptor dimerisation or clustering was proposed to be sufficient to initiate the catalytic activity of many RTKs. This is supported by the finding that antibody-mediated clustering (two epitope IgG binding to two receptors) of some RTKs was sufficient to promote their activation (Davis et al., 1994). However, it is becoming increasingly evident that the precise control of signalling cascades is likely to be multifaceted. For instance, there are a large number of RTKs containing pseudokinase domains that lack conventional catalytic activity. The organisation of pseudokinases into homo-dimers or heterodimerisation with other PKs can regulate their active counterparts (Mace and Murphy, 2021). In addition, there are ‘*bona-fide*’ catalytically active kinases that have regulatory functions independent of their kinase activity (Shaw et al., 2014).

With the exception of kinase-associated receptors, the other four members (outlined above) are able to sense external stimuli through intrinsic catalytic activity/transactivation. Alternatively, kinase-associated receptors act through ligand/effector downstream pathways, including activation by non-receptor tyrosine kinases (NRTKs). The RTK family of ‘receptor’ kinases is the largest superfamily (20 classes) and contains a range of receptors that regulate cell differentiation, proliferation, survival, metabolism, and migration. RTKs utilise several signalling pathways, including MAPK/ERK, PI3K/Akt/mTOR, PLCG1/PKC, JAK/STAT, Ras/Raf, Rac/MEK, and NF-κB. Interestingly, RTK signalling occurs in endosomes and at the cell surface, with different signalling pathways recruited depending on the location of the receptor. For kinase-associated receptors to propagate a signalling cascade, it is thought that several distinct processes must occur: (1) ligand binding, (2) activation of the intrinsic kinase domains, and/or (3) binding and activated NRTK to cell signalling (potentially through effectors). Despite the structural differences between kinase-associated receptors (e.g. cytokine receptors) and other intrinsically activated RTKs, the mechanism of ligand binding and re-organisation appears to be somewhat conserved. There are two major, distinct concepts to explain the activation of transmembrane and cell-surface receptors. Ligand binding induces either (1) dimerisation of receptors or (2) rearrangement of constitutively preformed dimeric receptors. A recent study seeking to investigate how two pseudokinases in the Eph family (EphB6 and EphA10) regulate signalling despite lacking kinase activity, found key intracellular residues that mediated the interaction of the kinases with SH2 domains of effectors for downstream signalling (Liang et al., 2021). The role of kinases extends far beyond phosphorylation, and non-catalytic activity, such as allosteric regulation, effector binding and scaffolding roles, are fundamental to biological mechanisms and signal transduction (Kung and Jura, 2016). The catalytic activity of EGFR receptors (human protein often referred to as ‘HER1/2’) is regulated by their interaction with the pseudokinase family member HER3. In a study combining the SMLM method dSTORM with protein cluster analysis, lapatinib-bound HER2 was found to form a complex with HER3 via a non-canonical kinase dimer structure that induced the formation of higher-order oligomers (Roberts et al., 2018).

### PK clustering via association with plasma membrane receptors

**Figure 4.**
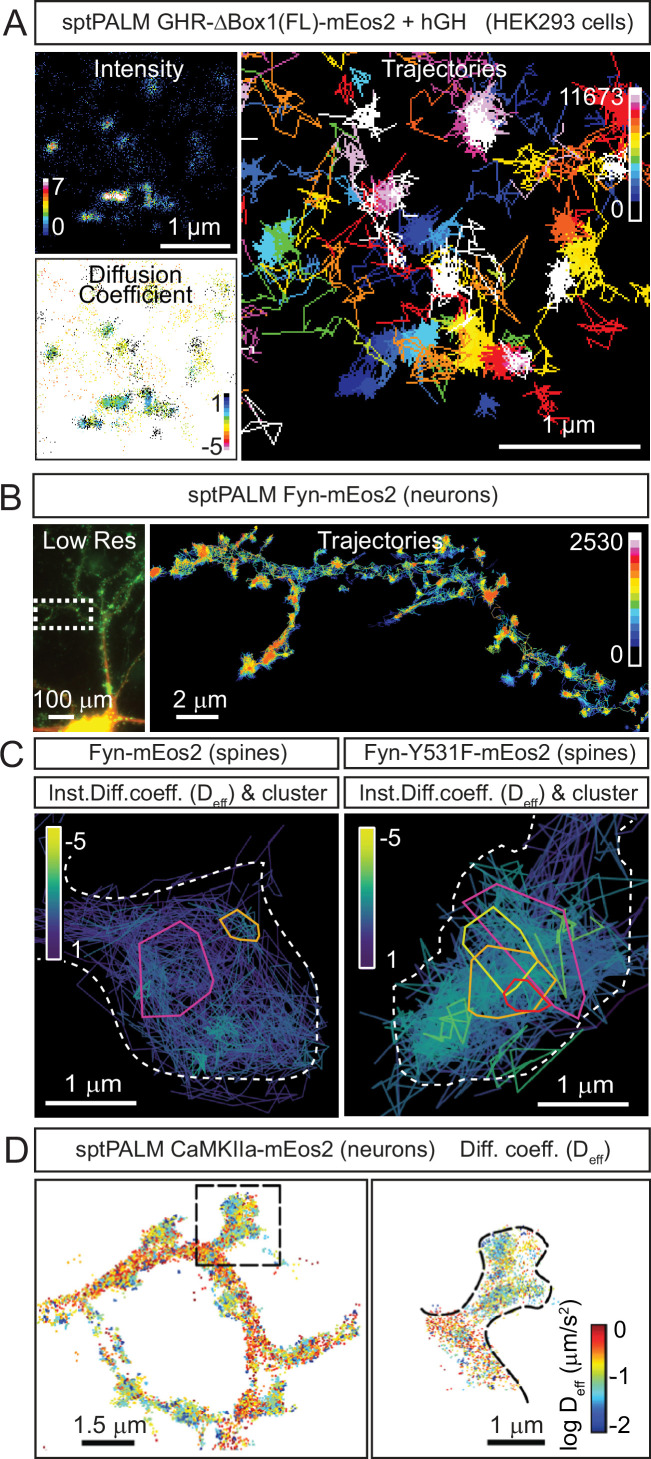
Examples of cellular nanoscale organisation of protein kinases. re(**A**) Representative sptPALM images of growth hormone receptor tagged with mEos2 fluorescent protein (GHR-Δ1(FL)-mEos2) expressed in HEK293 cells. Cells were incubated with human growth hormone (hGH) during the imaging. The panels show the high-resolution intensity map (Intensity), the diffusion coefficient map (Diffusion coefficient), where warmer colours represent lower mobility, and the trajectory map (Trajectories) where warmer tracks appear later in the acquisition. Images modified from Figure 6R-T of Chhabra et al., 2023. (**B**) Representative sptPALM images of Fyn-mEos2. The panels show the low-resolution epifluorescence image of a hippocampal neuron expressing Fyn-mEos2 (green) and mCardinal (red) acquired before the photoconversion of mEos2 molecules. The inset is shown at higher magnification in the right panel, where trajectories of single Fyn-mEos2 molecules can be observed. SptPALM imaging and analysis was performed in Martínez-Mármol et al., 2023b. (**C**) Representative sptPALM images of Fyn-mEos2 (left panel) or Fyn-Y531F-mEos2 (right panel) forming nanoclusters in dendritic spines. NASTIC analysis was used for the spatiotemporal distribution of single Fyn-mEos2 trajectories into nanoclusters. Individual trajectories were coloured based on their instant diffusion coefficients (*D*_eff_), with more immobile trajectories depicted in light colours and more mobile trajectories in dark colours. Panel modified from Figure 3B and C in Martínez-Mármol et al., 2023b. (**D**) Representative sptPALM images of CamKIIα-mEos2 in neurons. Single CamKIIα-mEos2 localisations were plotted into a diffusion coefficient map (*D*_eff_) where warmer colours represent higher mobility. The inset shows a dendritic spine at higher magnification in the left panel.

The cytokine receptor, growth hormone receptor (GHR) has recently been shown to form nanoclusters at the plasma membrane (Chhabra et al., 2023; Figure 4A). This clustering has been shown to be highly dependent on the competitive binding of two PKs with distinct downstream signalling (Chhabra et al., 2023). It is unclear whether these clusters are fostering the formation of dimers (Brooks et al., 2014), which is needed for effective signalling (Figure 1B). In the absence of ligand, GHR-ICD is in an inactive conformation. Structural changes in the GHR extracellular domain (ECD) induced by the ligand results in altered ratio of JAK2/STAT5 to ERK1/2 signalling, which has also been shown for other receptors, including the prolactin receptor (PRLR) and erythropoietin receptors (EpoR) (Rowlinson et al., 2008; Liu et al., 2009; Moraga et al., 2015; Zhang et al., 2015). The impact of the competitive binding of PKs for the GHR receptor nanoscale organisation and two alternative signalling pathways have recently been demonstrated (Chhabra et al., 2023). Upon growth hormone (GH) addition and Lyn binding/activation (ERK1/2 pathway), the GHR redistributes into larger clusters on the plasma membrane. Conversely, the activation of the JAK2/STAT pathway did not alter the distribution of the GHR within clusters. A recent study using single-molecule imaging to co-track the movement of monomeric GHR and its effectors showed that JAK2 was important in contributing to GHR dimerisation at the membrane (Wilmes et al., 2020). GHR is able to signal through at least two divergent NTRK pathways, (1) the JAK2/STAT and (2) the LYN/ERK1/2 pathway (Chhabra et al., 2021). Since the activation of GHR through the LYN/ERK pathway correlates with increased GHR endocytosis and degradation, its overall membrane organisation may be a key determinant by which a signalling cascade is initiated. In a similar line of research, the cytokine receptor interleukin-2 receptor gamma (IL-2Rγ) rarely exist as monomers, and cluster membership increased in response to IL-2 ligand stimulation. In addition, three distinct cluster sub-populations have been described: small ‘active’ clusters, medium clusters associated with endocytosis, and large ‘static’ clusters (Salavessa et al., 2021). Perturbation of either lipid rafts or F-actin increased the size of clusters to a similar level, but differentially affected signalling. Cholesterol depletion promoted assembly and sustained STAT5 and ERK signalling, whereas F-actin disruption blocked signalling all together. However, a live cell single-molecule imaging study on the cytokine receptor for thrombopoietin (TpoR) identified that in the absence of Tpo the ratio of monomer–dimer receptors was in equilibrium, and the presence of Tpo did not significantly perturb this ratio, but prolonged the stability of dimers. This study did not extend to investigate the clustering of TpoR (Sakamoto et al., 2016). Another single-molecule tracking study indicated that EGFRs reside outside lipid rafts in the absence of ligand but move into raft microdomains upon EGF binding (Lin et al., 2016). The pseudokinases PEAK1/3 are important scaffolding regulators for the EGFR signalling (Paul et al., 2022). A recent study investigating the PEAK3 interactome revealed that it acts as a dynamic scaffold together with essential adaptor proteins to regulate signal transduction (Roy et al., 2023). Lipid raft environment has been shown to enhance LYN kinase activity (Young et al., 2003). It is, therefore, likely that the presence of clusters, their size and sub-membrane distribution in regions with different lipidic composition, such as cholesterol-enriched microdomains, jointly act to mediate a tight control over the initiation and duration of signalling events. Pseudokinases likely play an integral role in the organisation and propagation of signalling hubs, yet it is a field that has not been fully explored using super-resolution approaches.

### Compartmentalisation of PKs in cellular organelles

Endosomal trafficking is critical for a multitude of cellular functions and includes transport of various RTKs such as EGFR and platelet-derived growth factor receptor and PKs associated with receptors such as GHRs (Sandilands and Frame, 2008). Importantly, upon cell activation, Src kinases were shown to translocate from the perinuclear region to the plasma membrane region on endosomes. An interesting concept is emerging, posing that RTK-containing endosomes could act as signalling units (or ‘quanta’), delivering their information to other subcellular destinations (Figure 3C). This has also been described as a form of analogue-to-digital conversion. This was demonstrated with the EGFR in fibroblasts (Villaseñor et al., 2015) and with the tyrosine receptor kinase B (TrkB) in hippocampal neurons (Wang et al., 2016a). The function of this type of endosomal signalling can differ. For instance, in neurons, axonal retrograde transport of signalling endosomes from the nerve terminal to the soma underpins neuronal survival. Each signalling endosome therefore carries a quantal amount of activated receptors, and it is the frequency of these retrogradely transported endosomes reaching the soma that determines the scale of the neurotrophic signal (Wang et al., 2016a). Notably, this study demonstrated that pharmacological and genetic inhibition of TrkB activation interfered with the coupling between synaptic activity and retrograde flux of signalling endosomes, suggesting that TrkB activity encodes for the level of synaptic activity experienced distally at nerve terminals and ‘digitalises’ it as a flux of retrogradely transported signalling endosomes. The ability of endosomes to signal is not limited to PKs and has also been shown for G-protein-coupled receptors (Irannejad et al., 2013). Therefore, endocytic trafficking of PKs likely represents a general mechanism inherent to the ability of endosomes to generate discrete signalling output (Murphy et al., 2009). Furthermore, PKs action is not limited to signalling through endocytic pathways. Indeed, the Src kinase has been shown to be critically involved in the anterograde pathway by controlling the recruitment of secretory vesicles to the cortical actin network in neurosecretory cells via direct anchoring of Myosin VI (Papadopulos et al., 2013; Tomatis et al., 2013).

### Subcellular membrane localisation of NRTKs via lipidation

One of the most studied mechanisms to control the organisation and function of kinases is modulating their binding affinity to biological membranes. NRTKs are unique, in that their subcellular location is heavily reliant on their conformation, which can be further influenced by a range of PTMs. NRTKs are categorised into nine subfamilies based on sequence similarities, primarily within the kinase domains. These include ABL, FES, JAK, ACK, SYK, TEC, FAK, CSK, and the SFKs. SFKs are active mediators of signal transduction pathways that influence cell proliferation, differentiation, apoptosis, migration, and metabolism. The SFKs members include Src, Lck, Hck, Blk, Fgr, Lyn, and Yrk. All SFKs contain modular Src homology domains (SH2, SH3, and SH4) at their N-terminus, a tyrosine kinase domain (SH1) and an intrinsically disordered region (IDR) at their C-terminus (Figure 3D). The intramolecular interaction of SFKs SH domains has been shown to mediate two main protein conformations: (1) open and (2) closed (when interacting). PTMs identified to influence these cytoplasmic receptors include ubiquitination, phosphorylation, and lipidation (Ortiz et al., 2021). Lipidation is essential to facilitate membrane attachment to those peripheral kinases that do not contain membrane-spanning domains. Lipidation consists of the covalent binding of specific lipid moieties to the protein body. Kinase lipidation can be performed co- or post-translationally, where at least two types of lipids have been described attached to kinases, including different forms of fatty acids and lipid-derived electrophiles (LDEs).

PK localisation and biological function can be regulated by the addition of two types of saturated fatty acyl chain, either the 14-carbon myristic acid (C14:0) or the 16-carbon palmitic acid (C16:0). Depending on the type of lipid incorporated, the fatty acylation can be classified as *N*-myristoylation or *S*-palmitoylation, respectively.

*N*-Myristoylation is an irreversible co-translational protein modification catalysed by *N*-myristoyl transferases (NMTs), where the fatty acid myristate is covalently attached to the N-terminal glycine of a protein. Both human NMT isozymes, NMT1 and NMT2, are expressed in most tissues, and have been implicated in the development and progression of diseases including cancer (Selvakumar et al., 2007), epilepsy (Selvakumar et al., 2005), Alzheimer’s disease (Su et al., 2010), and Noonan-like syndrome (Cordeddu et al., 2009). Examples of myristoylated kinases and their function can be found in Table 1, being SFKs some of the most studied lipid-modified kinases. In fact, all members of the SFK family are co-translationally myristoylated at the second glycine (Resh, 1994). Myristoylation is essential to anchor kinases in the cytoplasmic face of the plasma membrane and regulate their enzymatic activities, with significant consequences for the organism. A high-fat diet favours myristoylation and overactivation of the c-Src kinase, which accelerates xenografted prostate tumour growth in mice in vivo (Kim et al., 2017). Myristoylation also regulates kinase protein levels. Mutant c-Src lacking myristoylation showed reduced kinase activity but had enhanced stability, as its degradation by ubiquitination was diminished. This effect could be associated with the ability of the myristate group to facilitate the targeting of c-Src to the membrane, or to an intrinsic requirement for the myristate lipid in promoting ubiquitination and degradation by the E3 ligase Cbl (Patwardhan and Resh, 2010). Whereas myristoylation positively regulates most c-Src kinase activity, there are exceptions, such as the blockade of c-Abl tyrosine kinase by the addition of a myristoyl group. Under resting conditions, c-Abl is in an inactive state, which is maintained by the binding of the N-terminal myristoyl group to a hydrophobic pocket in the C-lobe of the kinase domain (Hantschel et al., 2003).

**Table 1.** Lipidation types of protein kinases.

Kinase	Type of phosphorylation	Role	Type of lipidation	Ref.
cAMP-dependent kinase (PKA)	Serine/threonine phosphorylation	Multiple roles in metabolism.	Myristoylation	Carr et al., 1982
AMP-activated protein kinase (b subunit) (AMPK)	Serine/threonine phosphorylation	Cellular energy (regulatory subunit).	Myristoylation	Oakhill et al., 2010
cGMP-dependent kinase II (PKGII)	Serine/threonine phosphorylation	Regulation of ion transport systems and nitric oxide levels.	Myristoylation	Vaandrager et al., 1996
P21-activated kinase 2 (PAK2)	Serine/threonine phosphorylation	Cytoskeleton re-organisation and nuclear signalling.	Myristoylation	Vilas et al., 2006
Casein kinase 1g (CK1g)	Serine/threonine phosphorylation	Antero-posterior patterning during development.	Myristoylation	Kinoshita-Kikuta et al., 2020
Protein serine kinase H1 (PSKH1)	Serine/threonine phosphorylation	Compartmentalisation of splicing factors.	Myristoylation and palmitoylation	Brede et al., 2003
Adenylate kinase 1 (AK1)	Adenosine diphosphate (ADP) phosphorylation	Cellular energy and homeostasis of adenine nucleotide ratios.	Myristoylation	Tomasselli et al., 1986
Blk (B lymphocyte kinase)	Tyrosine phosphorylation	B-cell receptor signalling and development; insulin synthesis and secretion.	Myristoylation	Resh, 1994
Fgr (Gardner-Rasheed feline sarcoma viral oncogene homolog)	Tyrosine phosphorylation	Cell migration and adhesion.	Myristoylation and palmitoylation	Resh, 1994
Hck (hematopoietic cells kinase, p59)	Tyrosine phosphorylation	Inflammatory response (cell survival and neutrophil migration).	Myristoylation and palmitoylation	Resh, 1994
Src	Tyrosine phosphorylation	Regulation of embryonic development and cell growth.	Myristoylation	Resh, 1994
Fyn (p59)	Tyrosine phosphorylation	T-cell differentiation; oocyte maturation; neuronal migration; myelination; synaptic function.	Myristoylation and palmitoylation	Koegl et al., 1994
Lck (T-cell-specific kinase, p56)	Tyrosine phosphorylation	Initiation of TCR signalling; T-cell development and homeostasis.	Myristoylation and palmitoylation	Koegl et al., 1994
Lyn (lymphocytes)	Tyrosine phosphorylation	Myeloid lineage proliferation; liver regeneration; osteoclast differentiation.	Myristoylation and palmitoylation	Resh, 1994
Yes (Yamaguchi sarcoma homolog, p61)	Tyrosine phosphorylation	T-cell migration; cancer cell proliferation and invasion.	Myristoylation and palmitoylation	Koegl et al., 1994

*S*-Palmitoylation (or *S*-acylation) is another type of fatty acylation, in which the fatty acid palmitate is covalently attached to cysteine residues. Contrary to myristoylation, protein palmitoylation happens post-translationally and is a reversible process catalysed by a large family of enzymes known as protein acyl transferases (DHHC-PATs) (Rana et al., 2019). The labile nature of the thioester bond makes palmitoylation a reversible and very dynamic process, where deacylation is performed by acyl protein thioesterases (APTs) or by lysosomal palmitoyl protein thioesterases (PPTs). The regulated activity of DHHC-PATs and APTs or PPTs facilitates a rapid turnover of membrane-bound palmitoylated proteins that can undergo cycles of acylation and deacylation in response to upstream signals (Rocks et al., 2005). Palmitoylation can also modulate the trafficking of the kinases. Mutant forms of Fyn lacking two palmitoylatable cysteines (cysteines 3 and 6) showed altered neuronal distribution, being unable to reach the dendritic arbour (Xia and Götz, 2014). Palmitoylation is usually found in combination with other lipid modifications. Several SFKs, including Fyn, Lyn, Lck, and Yes undergo both myristoylation and palmitoylation (Table 1). Whereas myristoylation alone facilitates the targeting of proteins to discrete membrane compartments, plasmalemma association of sole-myristoylated proteins is only transient with very short half-lives. The combination with palmitoylation mediates a stronger and longer membrane association. A peptide probe comprising the amino-terminal myristoylated and palmitoylated heptapeptide of the Fyn kinase showed an anterograde transport with initial Golgi accumulation before reaching the plasma membrane. Whereas palmitoylation was detected only on the Golgi, accelerating the anterograde transport of acylated targets, depalmitoylation occurred everywhere in the cell. The plasma membrane localisation of the peptide probe at steady state was more pronounced than other probes due to lower palmitate turnover kinetics (Rocks et al., 2010). Similarly, newly synthesised myristoylated Lyn and Yes initially enter the Golgi system, where they become palmitoylated, providing necessary access to the membrane secretory transport pathway *en route* to the plasma membrane, where Rab11 is involved (Sato et al., 2009). Deletion of three base pairs in the *Zdhhc21* gene resulted in the ‘depilated’ phenotype (*dep*), characterised by a variable hair loss, with thinner and shorter hairs remaining. This single mutation resulted in the loss of a highly conserved phenylalanine at position 233, causing the mislocalisation and loss of catalytic activity of the ZDHHC21 acyl transferase. Reintroducing wildtype ZDHHC21 into the mice rescued the shiny and smooth coat phenotypes. Fyn is a substrate for ZDHHC21, and the lack of palmitoylation caused altered Fyn localisation and downstream signalling activity, which resulted in reduced levels of Lef1, nuclear β-catenin, and Foxn1, altering keratinocyte differentiation, leading to hair loss (Mill et al., 2009). Overall, these results indicate that modulation of de-*A*cylation/re-*A*cylation cycles is an important mechanism that controls spatially and temporally the activity of SFKs, hence regulating a multitude of downstream signalling cascades with direct consequences in vivo.

Other types of lipidation can also occur, such as LDE modification of the ZAK kinase. LDEs are reactive lipid metabolites generated by lipid peroxidation when cells are under oxidative stress conditions. One important example of LDE is 4-hydroxy-2-nonenal (4-HNE), which is formed as a secondary intermediate by-product of lipid peroxidation (Ayala et al., 2014). 4-HNE is a reactive molecule that participates in multiple physiological processes as a nonclassical secondary messenger and can be covalently attached to numerous proteins, including the ZAK kinase (Wang et al., 2014b). This kinase is a member of the mitogen-activated protein kinase kinase kinase (MAP3K) family of signal transduction proteins, activating all three major MAPK pathways in mammalian cells (ERK1/2, JNK1/2/3, and p38 MAPK) (Yang et al., 2010b). The ZAK kinase is involved in the cellular response to UV radiation (Wang et al., 2005) and to chemotherapeutic agents (Wong et al., 2013). 4-HNE binds to ZAK in the conserved cysteine 22. The proximity of this position to the ATP-binding loop of ZAP, resulted in a 4-HNE-dependent blockade of ATP binding and loss of kinase activity. This creates a negative feedback mechanism that suppresses the activation of JNK apoptotic pathways induced by oxidative stress.

### Biomolecular condensates: a new frontier in PK organisation

Cytosolic PKs can also be organised in small clusters occurring outside the context of the plasmalemma or organellar membranes, within subcellular structures commonly referred as biomolecular condensates (BMCs) (Martínez-Mármol et al., 2023b). This type of membraneless compartmentalisation is characterised by the ability to concentrate charged and highly disordered molecules such as proteins and nucleic acids that undergo liquid–liquid phase separation (Banani et al., 2017). BMCs are stabilised by weak interactions between or IDRs of constituent molecules, creating a unique environment for the proteins contained inside. Since the probability of binding between two interacting molecules increases with the square of the binding density (Lagerholm and Thompson, 1998), BMCs are ideally positioned to host and/or initiate highly efficient cellular signalling. These phase-separated condensates are increasingly viewed as critical in a multitude of biological contexts. BMCs can also interface with the plasma membrane and generate hybrid systems, formed through the interplay between condensates and membrane constituents, such as transmembrane surface receptors. The Src kinase Fyn was recently shown to form nanoclusters in dendritic spines (Figure 4B, C) that are controlled by the ability of Tau to form BMCs (Martínez-Mármol et al., 2023b). As Fyn kinase binds to a number of post-synaptic proteins critically involved in synaptic plasticity, it is tempting to speculate that the integration of the signalling and plastic response could be regulated by nanoscale BMCs. Further protein phosphorylation and dephosphorylation can, in turn, modulate the fabric of these condensates, thereby generating multi-layered signal regulation. A recent study mapped a large number of phosphosites enriched within purified condensates, finding certain phosphosites modulate their ability to populate their condensates (Longfield et al., 2023). Ca^2+^/calmodulin-dependent protein kinase II (CaMKII) is a postsynaptic kinase that acts as a protein cross-linker, segregating synaptic molecules through Ca^2+^-dependent liquid–liquid phase separation formation (Hosokawa et al., 2021). CaMKII forms nanodomains in dendritic spines (Figure 4D; Lu et al., 2014). These nanodomains are essential for the establishment of trans-synaptic nanocolumns (Tang et al., 2016), which may be involved in establishing an optimal spatial arrangement between postsynaptic receptors and the location where neurotransmitters are released, a key mechanism for neuronal communication and synaptic plasticity. The composition, structure, formation, and role in signalling of BMCs are the subject of intense scrutiny. It is interesting to note that most in cellulo works involve large BMC structures amenable to fluorescent recovery after photobleaching. Classically, recovery from photobleach BMC is much slower than from the cytosol. In most cases, these studies have been performed in the context of protein overexpression. Whether kinase residency within BMCs is physiologically relevant is under debate. Recent studies suggest that BMCs can affect the clustering of PKs in the nanoscale range (Martínez-Mármol et al., 2023b).

### Implications of altered PK clustering in pathologies

Dysregulated receptor and kinase signalling is a common mechanism driving cancer progression (Brooks and Putoczki, 2020; Du and Lovly, 2018). Both cholesterol content and modification of phosphoinositide lipids affect transmembrane receptor clustering and signalling (Salavessa et al., 2021; McGraw et al., 2014; Wang et al., 2014a; Yang et al., 2010a; Rahbek-Clemmensen et al., 2017; Heidbreder et al., 2012; Murphy et al., 2013). Clinical observations provide support for the importance of cholesterol in regulating cytokine receptor signalling. For example, hypercholesterolaemia is associated with the development of leukaemia and has been shown to amplify cytokine signalling in leukaemia cells and alter SFK activation (Oneyama et al., 2009; Wang et al., 2016b). In addition, chronic lymphocytic leukaemia patients show a survival benefit from cholesterol-lowering with statin drugs (McCaw et al., 2017). Cholesterol levels have also been implicated in regulating other diseases for which cytokine signalling plays a major role, such as rheumatoid arthritis, where elevated LDL cholesterolaemia correlates with increased disease progression. Treatment with statins shows anti-inflammatory effects and reduced disease symptoms (Li et al., 2018; Park et al., 2013). Depletion of membrane cholesterol, with the consequent perturbation of lipid raft microdomains, inhibits JAK activation by ligand binding to GHR and IL-7R (Tamarit et al., 2013; Sandoval-Usme et al., 2014).

Interactions of receptors with phosphatidylinositol-4,5-bisphosphate (PtdIns(4,5)*P*_2_) have been shown to be an important mediator of receptor clustering and signalling. For example, EGFR forms significantly larger and more abundant nanoclusters in the membrane of lung cancer cells, compared to normal lung epithelial cells. EGFR clusters are mediated by their interaction with PtdIns(4,5)*P*_2_, as PtdIns(4,5)*P*_2_ depletion disrupts EGFR plasma membrane clustering, impairing EGFR signalling. Residues in the EGFR juxtamembrane (JM) region mediate the interaction with PtdIns(4,5)*P*_2_ and mutation of these JM residues similarly disrupts clustering and EGFR signalling (Wang et al., 2014a). The cytokine receptors GHR and prolactin receptor (PRLR), as well as the associated JAK2, are known to have specific interactions with PtdIns(4,5)*P*_2_. Mutations of residues in the PRLR that interact with PtdIns(4,5)*P*_2_ impair receptor signalling (Araya-Secchi et al., 2023; Haxholm et al., 2015). Furthermore, Ras (small GTPase) nanoclustering has been shown to be important in oncogenic signalling and attempts to disrupt Ras nanoclusters are being explored as a potential therapeutic strategy (Van et al., 2021). Several studies have identified Ras interaction with phosphatidylserine in membrane clustering, and a recent super-resolution microscopy study defined the nanoscopic spatial membrane association between Ras and phosphatidylserine (Koester et al., 2022).

SFKs are aberrantly activated in cancer, particularly in solid tumours (Martinsen et al., 2022). SFKs have been shown to play important roles in the clustering and mobility of receptors, such as nicotinic acetylcholine receptors, GPI-anchored receptors, B-cell receptors, and cytokine receptors (Chhabra et al., 2023; Flynn and Syed, 2020; Sohn et al., 2008; Suzuki et al., 2007; Stone et al., 2017). Increased membrane-saturated fatty acids induce c-Src activation and clustering within membrane subdomains. This was postulated to be a contributing mechanism that associates obesity with the development of type 2 diabetes (Holzer et al., 2011). Increased membrane cholesterol suppresses the ability of SFKs to induce cell transformation in fibroblasts, by redistributing these enzymes into cholesterol-enriched membrane microdomains, and sequestering them away from transforming signalling pathways (Oneyama et al., 2009). Proliferation of myeloproliferative neoplasms is commonly due to constitutive signalling of the cytokine receptor TpoR (MPL) (Murphy et al., 2013).

Since PKs play a major role in so many biological functions, alterations in these proteins play a particularly significant role in the progression of cancer, infectious diseases, and neurological disorders. The dysregulation of RTKs has been proposed for almost all forms of human cancer since they play such a major role in cell division. The tight regulation of these catalytically active receptors is key to regulating unwarranted cell division and tumourigenesis (Casaletto and McClatchey, 2012). In addition, the JAK/STAT signalling pathway is an intrinsic driver for metastasis (Brooks and Putoczki, 2020).

### PK nanoclustering for optimal output signal

**Figure 5.**
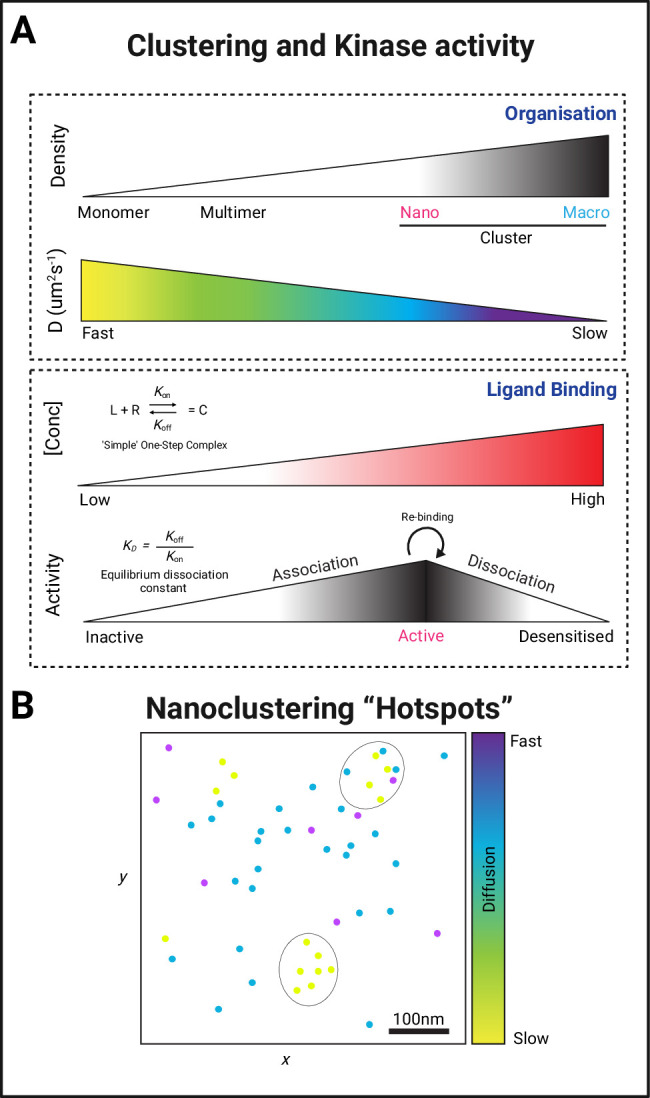
Forming protein kinase clusters. (**A**) PK clustering occurs via lateral trapping in nanoclusters that restrict their diffusion. In addition, the concentration of both ligand and receptor/substrate availability and the dissociation metrics of their interaction have been described to play a key role in determining the signalling strength of this biological event. (**B**) The regulation of both the size and location of clusters is therefore likely to determine the signalling duration through the creation of a nano-environment (circular areas containing a higher density of slow diffusing PKs) conducive for fast re-binding of proteins with their substrates and regulated signalling amplification through the creation of hubs that allow efficient effector association.

The binding affinity of ligands to their targets, such as a ligand to a cell-surface receptor, or a PK to its substrate is an important metric that ultimately controls signalling strength and duration. However, there are other factors that can make a substantial contribution to the signal output, such as local concentrations, protein conformational states, and molecular crowding. The terms affinity and avidity are often used in the context of antibody binding, but the principles of avidity/multivalency have also been applied to characterise several other types of interactions (Erlendsson and Teilum, 2020), such as inter-molecular interactions (allosteric proteins and IDPs) (Tompa, 2014), and interactions associated with short linear motifs (Bugge et al., 2020). Since receptors and associated PKs and pseudokinases can potentially participate in more than one signalling cascade, the mechanism by which they can favour one over the other is still mostly elusive. Other factors in determining the nature and strength of the output signal are (1) the probability of ligand/binding, activation and dissociation (Vauquelin and Charlton, 2010), (2) their multimeric state (Chhabra et al., 2023; Brooks et al., 2014), and (3) their diffusive and clustering properties (Small et al., 2020), and the local concentration (Caré and Soula, 2011; Figure 5). It has therefore been proposed that the residence of proteins within subcellular compartments, such as lipid rafts or BMCs, likely serves to modulate their activity through dynamic clustering mechanisms (Erlendsson and Teilum, 2020). More experimental and in silico work are needed to elucidate the precise cellular organisation of PKs to optimally amplify and locally control signalling. Since the precise organisation of PKs can now be explored using various super-resolution microscopy methods, much can be learnt about the mechanistic details of PK signalling. For instance, how the discrete signalling hubs at the membrane are formed and maintained, and how they are affected by various ligands and effectors. Furthermore, by applying SPT, some key questions in PK biology will begin to be answered, such as (1) is PK organisation affected during cancer progression and metastasis; (2) how do protein effectors modulate signalling strength; and (3) how do PKs differentiate alternative down-stream signalling pathways. Furthermore, the nanoscale distribution of pseudokinases (or ‘kinase-dead’ variants), and their effect on binding partners and effectors (including active PK binders) will require further investigation. The ability of a single type of receptor to activate numerous signalling responses likely relies upon these biophysical properties to modulate RTKs, kinase-associated receptors, non-receptor PKs, and pseudokinases.
